# Two Japanese patients with metastatic castration-resistant prostate cancer with somatic biallelic *BRCA2* loss and *RB1* splice site variant or loss who responded to Poly-ADP-ribose polymerase inhibitor: A case report

**DOI:** 10.1007/s13691-025-00761-2

**Published:** 2025-04-10

**Authors:** Shiori Miyachi, Takeshi Sasaki, Momoko Kato, Katsunori Uchida, Shunsuke Owa, Taketomo Nishikawa, Shinichiro Higashi, Hiroto Yuasa, Kouhei Nishikawa, Yoshinaga Okugawa, Masatoshi Watanabe, Takahiro Inoue

**Affiliations:** 1 Department of Nephro-Urologic Surgery and Andrology, Mie University Graduate School of Medicine, 2-174 Edobashi, Tsu, Mie 514-8507 Japan; 2 Department of Oncologic Pathology, Mie University Graduate School of Medicine, Mie, Japan; 3 Department of Pathology, Kansai Medical University, Osaka, Japan; 4 Department of Genomic Medicine, Mie University Hospital, Mie, Japan

**Keywords:** *BRCA2* loss, *RB1*, Prostate cancer, Japanese, Poly-ADP-ribose polymerase inhibitor

## Abstract

We treated two patients with metastatic castration-resistant prostate cancer (mCRPC) who achieved a response duration of more than 12 months with Poly-ADP-ribose polymerase inhibitor (PARPi). Case 1 was a patient in his 60s with lung metastases, and case 2 was in his 70s and presented liver metastases. Genetic tests (FoundationOne^®^ CDx) were performed. Both patients had somatic biallelic *BRCA2* loss, together with *RB1* splice site variant (NM_000321.3:c.2489 + 1G > C) or *RB1* loss. After PARPi administration, their metastatic sites had shrunk enough to keep partial response. These cases suggested that patients with mCRPC with biallelic *BRCA2* loss and the *RB1* splice site variant or loss may have remarkable response to PARPi.

## Introduction

*BRCA1/2* pathogenic variants are the most common variants in DNA damage repair genes related to prostate cancer (PCa) [1]. Poly-ADP-ribose polymerase inhibitor (PARPi) is a targeted therapy based on gene variants, especially *BRCA1/2* pathogenic variants, and has been shown to be effective in treating patients with metastatic castration-resistant prostate cancer (mCRPC) [2].

The PROfound trial examined the efficacy of the PARPi olaparib in patients with mCRPC who progressed on androgen receptor signaling inhibitors (ARSIs, i.e. enzalutamide or abiraterone) and taxane treatment. Image-based progression-free survival (rPFS) was 7.4 months in patients with *BRCA1*, *BRCA2*, or *ATM* variants [3]. In addition, rPFS at 12 months in the *BRCA1/2*, *ATM* cohort was 28%, which limited the number of patients who can be expected to achieve a long-term response to olaparib [3].

Olaparib is not effective in all cases of mCRPC with *BRCA1/2* pathogenic variants, and even if temporarily effective, drug resistance is acquired [2]. In this report, we describe our experience with two patients with mCRPC, with somatic biallelic *BRCA2* loss and a *RB1* splice site variant or loss, who achieved a response to olaparib treatment for more than 12 months.

## Case report

### Case 1

**Fig. 1:**
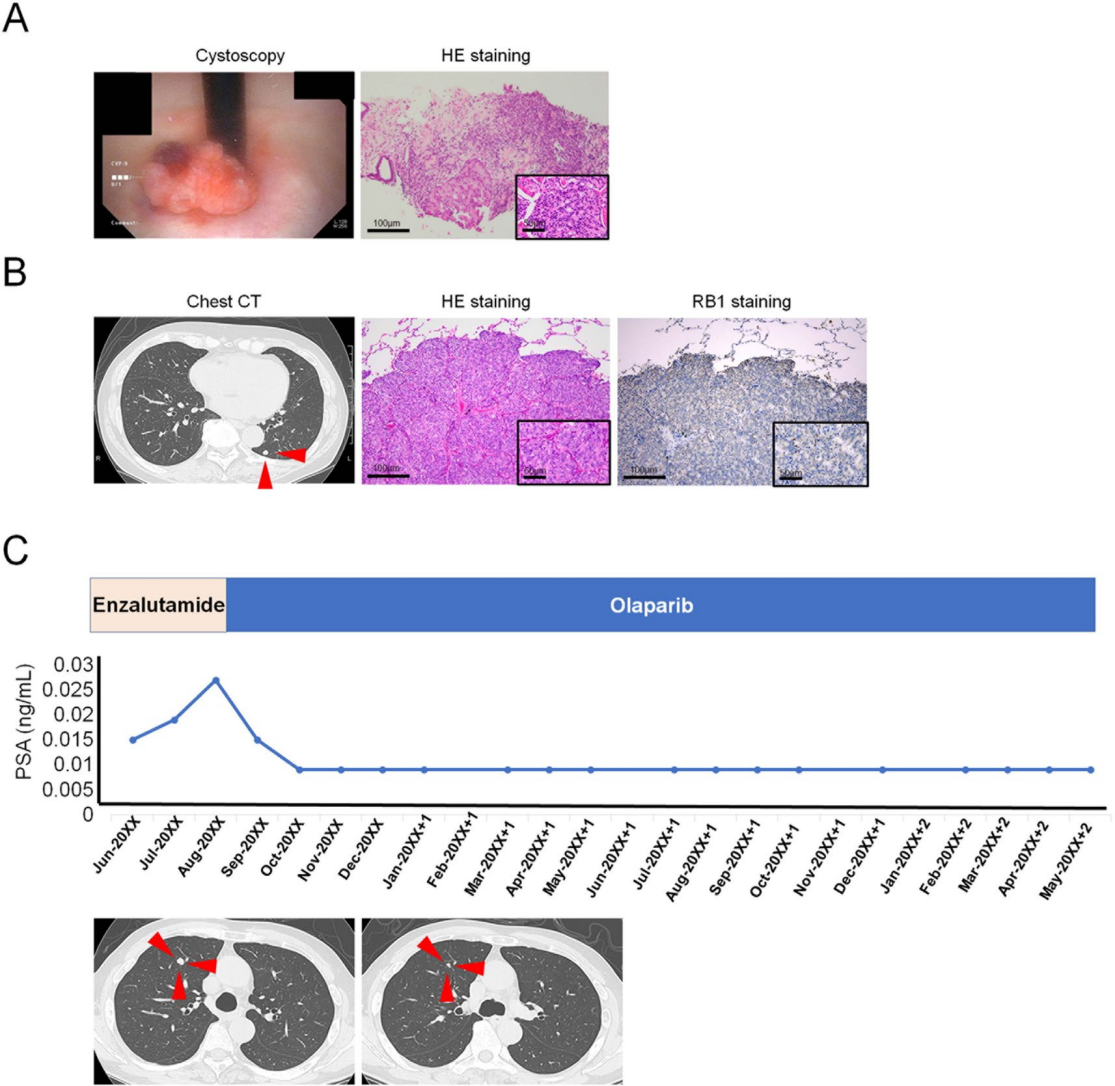
Case 1: clinical and pathological information Cystoscopy shows a bladder-neck tumor **A**. Histopathological examination indicates Grade Group 5 adenocarcinoma (scale bar = 100 µm) and intraductal carcinoma (high magnification, insert, scale bar = 50 µm) (A). Chest CT shows lung metastasis (red arrowheads) after combined androgen blockade and intensity-modulated radiation therapy (B). Histopathological examination reveals adenocarcinoma of lung metastasis (bar = 100 µm) and high magnification (insert, scale bar = 50 µm) **B**. Immunostaining for RB1 is negative in the lung resected specimens (scale bar = 100 µm) and high magnification (insert, scale bar = 50 µm) (B). Clinical course of the patients after diagnosis of mCRPC, showing administrated anti-tumor drugs and his PSA levels (C). *CT* Computed tomography, *PARPi* Poly-ADP-ribose polymerase inhibitor

A 69 year-old Japanese man presented with gross hematuria. He had no significant previous diseases or a family history of prostate, pancreatic, breast, and ovarian cancers. Cystoscopy revealed a bladder-neck tumor (Fig. 1A). A whole-body radiologic investigation showed no obvious metastases. The initial serum prostate-specific antigen (PSA) level (1.690 ng/mL) was within normal limits. We performed transurethral resection of bladder tumor and systematic prostate biopsy. Histopathological examination revealed Grade Group 5 adenocarcinoma and intraductal carcinoma (Fig. 1A). After combined androgen blockade (CAB) and intensity-modulated radiation therapy (IMRT), lung metastasis developed (Fig. 1B), leading to mCRPC. The lung metastasis was subsequently resected, and histological analysis confirmed it as adenocarcinoma, consistent with prostate cancer metastasis (Fig. 1B). Subsequently, ARSI (enzalutamide) was administered, but new lung metastases appeared. Genetic testing (FoundationOne^®^ CDx) of the resected lung metastasis specimens showed biallelic *BRCA2* loss, and *RB1* (NM_000321.3:c.2489 + 1G > C), *APC* (D1636fs*2, c.4906_4907insG), and *PTPRO* (S802fs*6, c.2403delC) variants. Interestingly, genetic testing (FoundationOne^®^ CDx) of prostate biopsy specimens at diagnosis and of resected lung metastatic tissues during mCRPC staging revealed the same genetic variants, indicating that these occurred in the primary site before treatment. Immunostaining for RB1 (Abcam ab14715, clone 2E3GC12FB2AE2, Cambridge, UK) was negative in the resected lung specimens (Fig. 1B). The patient started treatment with the PARPi olaparib and achieved a partial response in the lung metastases, maintaining this response for 24 months after starting PARPi treatment (Fig. 1C). Serum PSA levels decreased with treatment (Fig. 1C). The patient underwent clinical genetic counseling followed by family genetic testing [multiplex ligation-dependent probe amplification (MLPA) assay of *BRCA2*; NM_000059.3]. The patient was negative for *BRCA2* germline variants.

### Case 2

**Fig. 2:**
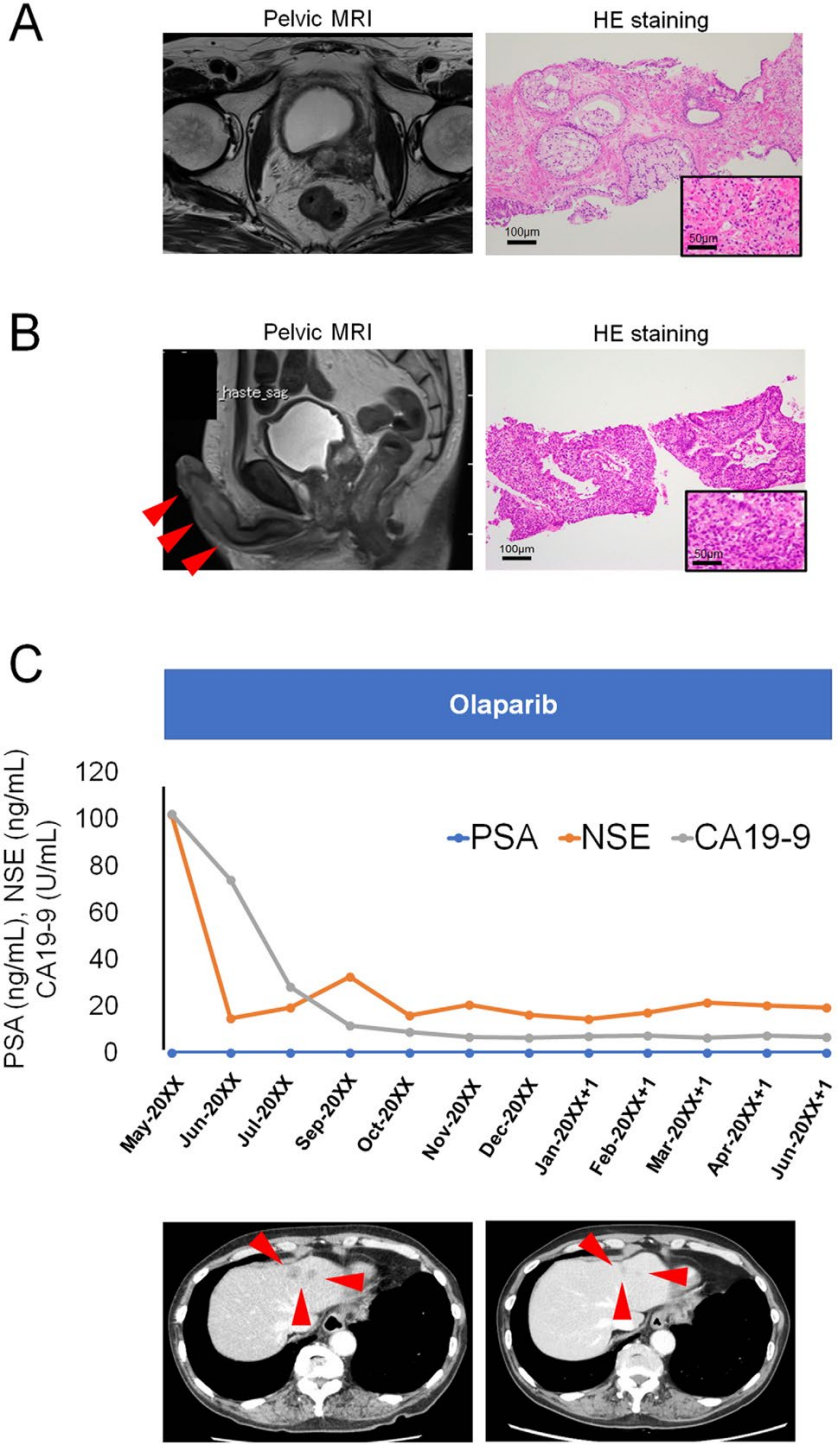
Case 2: clinical and pathological information Pelvic MRI shows localization of PCa in the left lobe of the prostate (A). Histopathological examination indicates Grade Group 5 adenocarcinoma (bar = 100 µm) and intraductal carcinoma (high magnification, insert, scale bar = 50 µm) **A**. Pelvic MRI shows priapism (red arrowheads) caused by penile metastasis during ARSI treatment **B**. Histopathological examination indicates Grade Group 4 adenocarcinoma of prostate re-biopsy specimens (bar = 100 µm) and high magnification (insert, scale bar = 50 µm) (B). The patient has been treated with the PARPi olaparib, and has maintained a partial response in liver metastases for 13 months after starting PARPi treatment (C). Serum PSA levels were below detectable range throughout, but serum NSE and CA19-9 levels decreased with treatment **C**. *MRI* Magnetic resonance imaging, *PCa* Prostate cancer, *ARSI* Androgen receptor signaling inhibitor, *PARPi* Poly-ADP-ribose polymerase inhibitor

A 73-year-old Japanese man presented with backache. He had no significant previous diseases or a family history of prostate, pancreatic, breast, and ovarian cancers. The initial serum PSA level was 48.25 ng/mL. Pelvic magnetic resonance imaging (MRI) revealed PCa in the left lobe of the prostate (Fig. 2A). A whole-body radiologic investigation showed bone and lymph node metastases. We performed systematic prostate biopsy. Histopathological examination revealed Grade Group 5 adenocarcinoma and intraductal carcinoma (Fig. 2A). During ARSI (abiraterone) treatment, gross hematuria and malignant priapism appeared without serum PSA level elevation (< 0.009 ng/mL), and a whole-body radiologic investigation showed penile and liver metastases resulting in mCRPC. Prostate re-biopsy specimens revealed viable adenocarcinoma, consistent with PCa (Fig. 2B). Neuroendocrine markers, e.g. chromogranin A and synaptophysin, were not stained. AR staining was weak (Supplemental Fig. 1). Genetic testing (FoundationOne^®^ CDx) of the prostate re-biopsy specimens showed biallelic *BRCA2* loss, biallelic *RB1* loss, *TP53* variant (V173A, c.518 T > C), CDC73 (L460fs*6, c.1379_1387TTTTGCCTG > C), *PIK3R1* variant (C569fs *2,c.1975_1978delTGCT), and *RAD51D* variant (K91fs*13, c.271_272insTA). The patient started treatment with the PARPi olaparib and achieved a partial response in the liver metastases, maintaining it for 13 months after starting PARPi treatment (Fig. 2C). Serum PSA levels have remained undetectable throughout, while serum NSE and CA19-9 levels decreased with treatment (Fig. 2C). The patient underwent clinical genetic counseling followed by family genetic testing [MLPA assay of *BRCA2*; NM_000059.3 and *RAD51D*; NM_002878.3]. The patient had *RAD51D* germline variants.

## Discussion

We treated two patients with mCRPC who achieved a response for more than 12 months with PARPi treatment. Both cases had not only *BRCA2* loss but also *RB1* variants (splice site variant or loss), and these double variants are known to lead to an aggressive phenotype with a epithelial to mesenchymal transition [4]. *BRCA2* loss may be less likely to cause reversion mutation, one of the mechanisms of PARPi resistance [5], and the two cases in this study are predicted to have long-term response to PARPi treatment. A recent report showed that PCa organoids harboring a heterozygous co-deletion of *BRCA2* and *RB1* alleles exhibited depletion of both BRCA2 and RB1 proteins, suggesting that heterozygous loss leads to haploinsufficiency of these proteins. Since the *RB1* variant (NM_000321.3:c.2489 + 1G > C) has been reported to disrupt the canonical splice site, potentially leading to aberrant splicing and either an abnormal protein or a transcript subject to nonsense-mediated mRNA decay, a similar process might have occurred in our case 1, resulting to co-deletion of *BRCA2* and *RB1* alleles. PCa with *BRCA2* loss and concomitant *RB1* co-loss has been reported as more sensitive to PARPi treatment than that with *BRCA2* loss alone [6]. This may be another reason why PARPi was effective in the long term in these two cases of *BRCA2* and *RB1* double variants. Although several *BRCA2* and *RB1* co-loss cases had been reported [7–10], to our knowledge, only one report showed the efficacy of PARPi (veliparib) in patients with mCRPC with *BRCA2* and *RB1* co-loss [7], as in our case (Table 1). However, Taza F et al. reported that PARPi were effective in 11 of 19 mCRPC patients with co-mutation of *RB1* and *BRCA2*, whereas PARPi were effective in 53 of 80 mCRPC patients without *RB1* but with *BRCA2* mutation[11].

**Table 1:** Case reports of PCa with *BRCA2* and *RB1* variants

Case	Age	Initial PSA (ng/mL)	Gleason score	TNM	Gene alterations
Romeo-Laorden N, et al. [10]	78	24.7	4 + 4	cT2xN0M0	*BRCA2*, *Rb1*
Izawa M, et al. [11]	62	19.8	4 + 5	cT3bN0M0	*BRCA2*, *Rb1*,* IDH, AR, MYC, ERBB2, ERBB3, ESR1, NRAS, SMO, AKT2*
Matsumoto K, et al. [12]	59	4.5	4 + 3	cT2aN0M0	*BRCA2*, *Rb1*, *APC*, *ATM*
Iwasawa T, et al. [13]	61	165.42	5 + 4	cTxNxM1b	*BRCA2*, *Rb1*, *TP53*, *AR*
Present case 1	69	1.69	5 + 4	cT4N0M0	*BRCA2*, *Rb1*, *APC*, *PTPRO*
Present case 2	73	48.25	4 + 5	cT3bN1M1b	*BRCA2*, *Rb1*, *TP53*, *CDC73*, *PIK3R1*, *RAD51D*

According to the ClinVar Database (https://www.ncbi.nlm.nih.gov/clinvar), the *RB1* variant (NM_000321.3:c.2489 + 1G > C) has been previously reported to result in a splice variant, potentially causing intron inclusion between exons 23 and 24. However, based on the COSMIC Database (https://cancer.sanger.ac.uk/cosmic), the amino acid change is unknown (Genomic Mutation ID: COSV57300119). The *RB1* variant (NM_000321.3:c.2489 + 1G > C) has been reported as a somatic variant in only one patient with metastatic PCa [12]. To check if the *RB1* variant resulted in RB1 protein loss as indicated in the previous report, we performed immunostaining for RB1, which was negative in lung resection specimens.

In case 2, besides *BRCA2* loss, biallelic *RB1* loss and *TP53* variant were present. The co-loss of *TP53* and *RB1* is known as one of the features of neuroendocrine prostate cancer, resulting in decreased AR expression and increased neuroendocrine marker expression[13]. Iwasawa et al. reported that *BRCA2* alteration was found only in the *TP53* and *RB1* altered CRPC cases without neuroendocrine differentiation[10]. In case 2, similarly, AR expression was low but neuroendocrine markers, e.g. chromogranin A and synaptophysin, were completely not stained by immunostaining.

Among *BRCA2* variants of PCa, biallelic homozygous deletions are common. Sokol et al. reported that 28.9% (176/609) of *BRCA2* variants of PCa were biallelic homozygous deletions (shown in Supplementary Table of Ref [14]). Both heterozygous and homozygous *BRCA2* losses had poor prognosis with non-PARPi treatment [15]. Conversely, patients with mCRPC with *BRCA2* homozygous deletion had superior rPFS and overall outcomes with PARPi treatment, suggesting that, in these tumors, PARPi resistance may be harder to develop [16].

In addition, there are two interesting aspects of these two cases. First, the two cases had IDCP genomic characteristics with distinct genomic profiles of somatic variants (*ERG* rearrangement, the loss or variants of *RB1*, *TP53*, and *PTEN*, as well as *MYC* amplification) and *BRCA2* variants [17]. Second, both cases had low serum PSA levels at the time of mCRPC, which may indicate the presence of pathogenic variants of *RB1* and/or *TP53* [18].

This report has several limitations. First, the mechanism of biallelic somatic *BRCA2* loss has not yet been elucidated. Second, it is not clear whether the *RB1* splicing variant in Case 1 is identical to *RB1* loss. Third, treatment with the PARPi olaparib is ongoing and it is not clear how long the therapeutic effect will continue.

In conclusion, these two cases of Japanese patients with mCRPC suggest that PARPi may be effective in the long term in patients with *BRCA2* and *RB1* double pathogenic variants. Further analysis is needed to evaluate if the presence of *RB1* mutation can be a predictive biomarker for the efficacy of PARPi in patients with BRCA-mutated mCRPC.

## Data Availability

The data support the findings of this study are available from the corresponding author upon reasonable request.
